# Inflammatory Factors in Early Stages of Psychosis

**DOI:** 10.1192/j.eurpsy.2025.143

**Published:** 2025-08-26

**Authors:** A. Catalan

**Affiliations:** OSI Bilbao-Basurto, Bilbao, Spain

## Abstract

**Abstract:**

Growing evidence suggests that inflammation plays a critical role in the early stages of psychosis, potentially contributing to disease onset and progression. Several studies have identified elevated levels of pro-inflammatory cytokines, such as interleukin-6 (IL-6) and tumor necrosis factor-alpha (TNF-α), in individuals at clinical high risk (CHR) for psychosis and in those with first-episode psychosis (FEP). However, inconsistencies across studies highlight the need for further research to clarify the relationship between immune dysregulation and psychosis onset.

This talk reviews the current literature on inflammatory markers in early psychosis and their potential implications for pathophysiology, early detection, and treatment strategies. Notably, inflammation may serve as a promising biomarker for identifying individuals at risk and monitoring disease progression. Additionally, anti-inflammatory interventions are being explored as potential adjunctive treatments for psychosis.

Beyond the review of existing evidence, we will present **original data** from the **PREGAP study** conducted at the **Early Intervention \Service (EIS) of Basurto University Hospital**. This dataset includes inflammatory marker assessments in CHR and FEP individuals, providing novel insights into immune alterations in early psychosis.

Understanding the role of inflammation in psychosis could open new avenues for personalized medicine, early intervention, and improved treatment outcomes. Future research should focus on large-scale longitudinal studies to confirm the clinical utility of inflammatory markers and identify potential therapeutic targets.

**Disclosure of Interest:**

None Declared

